# The inter-visit variability of retinal blood flow velocity measurements using retinal function imager (RFI)

**DOI:** 10.1186/s40662-018-0124-z

**Published:** 2018-12-04

**Authors:** Yuqing Deng, Meng Li, Gengyuan Wang, Hong Jiang, Jianhua Wang, Jing Zhong, Saiqun Li, Jin Yuan

**Affiliations:** 1 State Key Laboratory of Ophthalmology, Zhongshan Ophthalmic Centre, Sun Yat-sen University, Xianlie Road 54, Guangzhou, 510060 China; 2 Bascom Palmer Eye Institute, University of Miami Miller School of Medicine, Miami, FL USA

**Keywords:** Inter-visit variability, Retinal blood flow velocity, Retinal function imager, Concordance correlation coefficient (CCC), Coefficient of variance (CV)

## Abstract

**Background:**

To determine the inter-visit variability of retinal blood flow velocities (BFVs) using a retinal function imager (RFI) in healthy young subjects.

**Methods:**

Twenty eyes of 20 healthy young subjects were enrolled. RFI imaging was performed to obtain the BFVs in retinal arterioles and venules in a field measuring 7.3 × 7.3 mm^2^ (setting: 35 degrees) centered on the fovea, and repeated measurements were obtained on two separate days. The inter-visit variability of BFVs was assessed by the concordance correlation coefficient (CCC) and coefficient of variance (CV).

**Results:**

At the first visit, the mean BFV was 3.6 ± 0.8 mm/s and 3.0 ± 0.7 mm/s in arterioles and venules, respectively, which were not significantly different from those at the second visit (the BFV of arterioles was 3.5 ± 0.8 mm/s, and the BFV of venules was 3.0 ± 0.7 mm/s, *P* > 0.05, respectively). The CCC was 0.72 in the BFVs of arterioles and 0.67 in venules, and the CV was 10.8% in the BFVs of arterioles and 11.0% in venules.

**Conclusion:**

The inter-visit variability using the retinal function imager (RFI) with a large field of view appeared to be good and comparable to previously reported intra-visit and inter-eye variability.

## Background

Blood flow is important for maintaining the integrity of the retina. Altered blood flow may lead to retinal dysfunction and damage [1, 2]. Previous studies have also indicated that systemic diseases associated with the cardiovascular system give rise to a significant change in retinal blood flow [3–7]. Measuring the retinal microcirculation may give a better understanding of the pathogenesis of retinal diseases. Many methods have been applied to measure retinal blood flow. These methods for measuring retinal blood flow include laser Doppler flowmetry [8], ultrasound flowmetry [9] and fluorescence fundus angiography [10]. Flowmeters mainly measure the large vessels in the retina, such as central retinal arteries and veins, which may not reflect the changes in the microvasculature. Fluorescence fundus angiography requires the injection of dyes and the procedure is time-consuming, which prevents it from being widely used for measuring retinal blood flow.

The retinal function imager (RFI) (Optical Imaging Ltd., Rehovot, Israel) is an in vivo and non-invasive imaging device that applies high resolution and high-speed camera and spectroscopic illumination for directly measuring retinal blood flow velocity in small vessels. This rapid measurement does not require any external dyes because the hemoglobin in red blood cells is used as the intrinsic contrast [11, 12]. Since the camera captures frames at 50–60 Hz, the system is enhanced to visualize and extract subtle changes by recording the path of red blood cells from a series of 8 images in one shot and then calculate blood flow velocity. Measurements are synchronized with the heart beat using a probe attached to a finger to reduce the influence of the cardiac cycle on these measurements. The applications of RFI have been extensively covered in a recent review [13]. Using RFI, the retinal microcirculation has been studied in healthy subjects and patients with diseases, such as multiple sclerosis [14], diabetic retinopathy [15], glaucoma [16] and retinitis pigmentosa [17]. Segmental reproducibility, inter-class validity, and variability in the same day have been evaluated to validate the measurements made using RFI [18, 19]. However, the inter-visit variability has been tested only on a very small group. With the further widespread use of RFI in the clinic and research, additional investigations of the inter-visit variability are beneficial for the design of longitudinal studies, which require repeated measurements of the retinal microcirculation. The aim of this study was to determine the inter-visit variability of retinal blood flow velocities (BFVs) obtained using RFI in healthy young subjects.

## Methods

### Subjects

This study was approved by the Institutional Review Board of the Zhongshan Ophthalmic Center, Sun Yat-sen University, China, and adhered to the tenets of the Declaration of Helsinki. Informed consent was obtained from each of the enrolled subjects. Subjects without ocular abnormity and intraocular pressure (IOP) lower than 21 mmHg were included. Patients with systemic diseases, including diabetes, hyperlipidemia, and hypertension, refractive media opacity and high refractive error (more than − 6 diopters) were excluded. Twenty eyes of 20 individuals with no ophthalmic pathology were enrolled from April 4th to May 3rd in 2018. All participants underwent blood pressure and heart beat measurements as well as intraocular pressure (IOP) evaluation using an automated non-contact tonometer (TX-20, Full Auto Tonometer, Canon, U.S.A.).

### Retinal function imager (RFI) and imaging procedure

A Retinal Function Imager (RFI-3000, Optical Imaging, Rehovot, Israel) was used to measure retinal blood flow velocities; this system has been used in clinical research for more than 10 years [13]. Briefly, this imaging system is a fundus camera-based system that uses a high speed and high-resolution digital camera and a stroboscopic flash lamp power supply to capture the motion of the red blood cells in the retinal vessel. The inherited software tracks the red blood cells in a series of images for the measurement of retinal blood flow velocity. Per the manufacturer’s instructions, more than 4 series are needed, and at least 4 well-focused images are needed in each series [20]. A field of view of 7.3 × 7.3 mm^2^ was used, which was centered on the fovea. During RFI imaging, the subject’s pupil was dilated to at least 6 mm, and one randomly selected eye was measured from each subject.

### Image analysis

RFI imaging processing software (Browse ver.2.2.0.236) was used to measure blood flow velocity. Briefly, sophisticated algorithms were designed to track the erythrocyte movement by aligning and comparing a series of images. Calculation of the velocities was performed automatically by detecting the movement of hemoglobin as an optical marker. The vessels were manually selected by drawing the pathways. The second and tertiary branches of retinal vessels were selected. Once the vessels were selected, blood flow velocity was measured and averaged from this registered series of images.

**Fig. 1:**
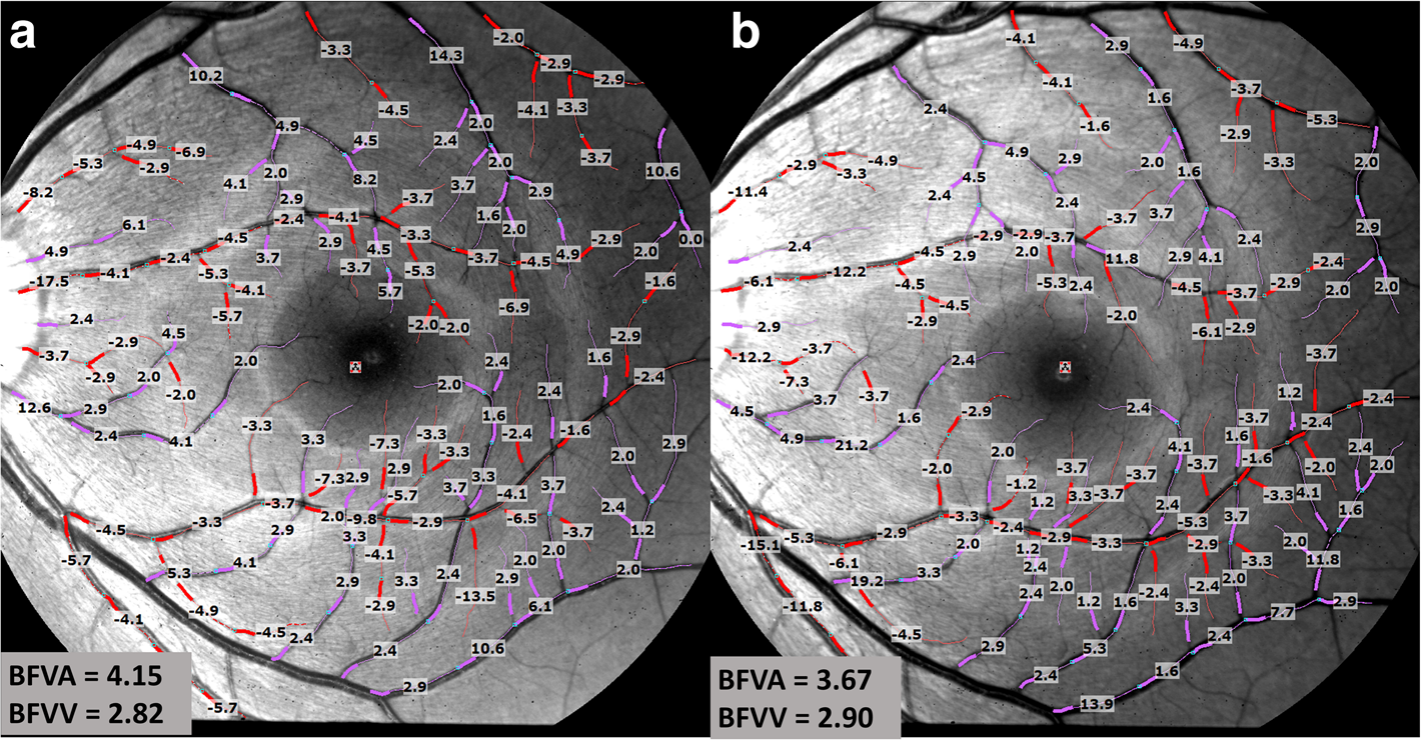
Repeated measurements of retinal blood flow velocities using the RFI. Two different sessions were scheduled on two different days involving the same location of the same eye (**a**: first visit, **b**: second visit). The arterioles are marked in red (BFVs as the negative values, flow towards the tissue), and the venules are marked in purple (BFVs as the positive values, flow leaving the tissue). BFVA = blood flow velocity of arterioles, BFVV = blood flow velocity of venules

The mean velocity and standard deviation were exported. If the standard deviation over the mean was more than 0.45, the vessel segment was removed because the measurement was considered unreliable per the manufacturer’s instructions [19]. Analysis of 2 measurements on two separate days was performed by one operator. The mean interval between visits were 5 days, and the differences in time of the two dates were 1.9 h. The results (mean and SD of BFV) were determined automatically by processing software (Fig. 1).

### Statistical analysis

Statistical analysis was performed using SPSS software, version 22 (IBM Inc., Armonk, NY). The inter-visit repeatability of blood flow velocity was assessed by the concordance correlation coefficient (CCC) and coefficient of variance (CV). Limits-of-agreement plots (Bland–Altman plots) were used to assess the difference between the measurements from two sessions on different days. Paired t-test was used to test the differences between the two visits. Nonparametric correlations were performed between RFI measurements and eye, age, sex, blood pressure, HR, and IOP. *P* values less than 0.05 were considered significant.

## Results

**Fig. 2:**
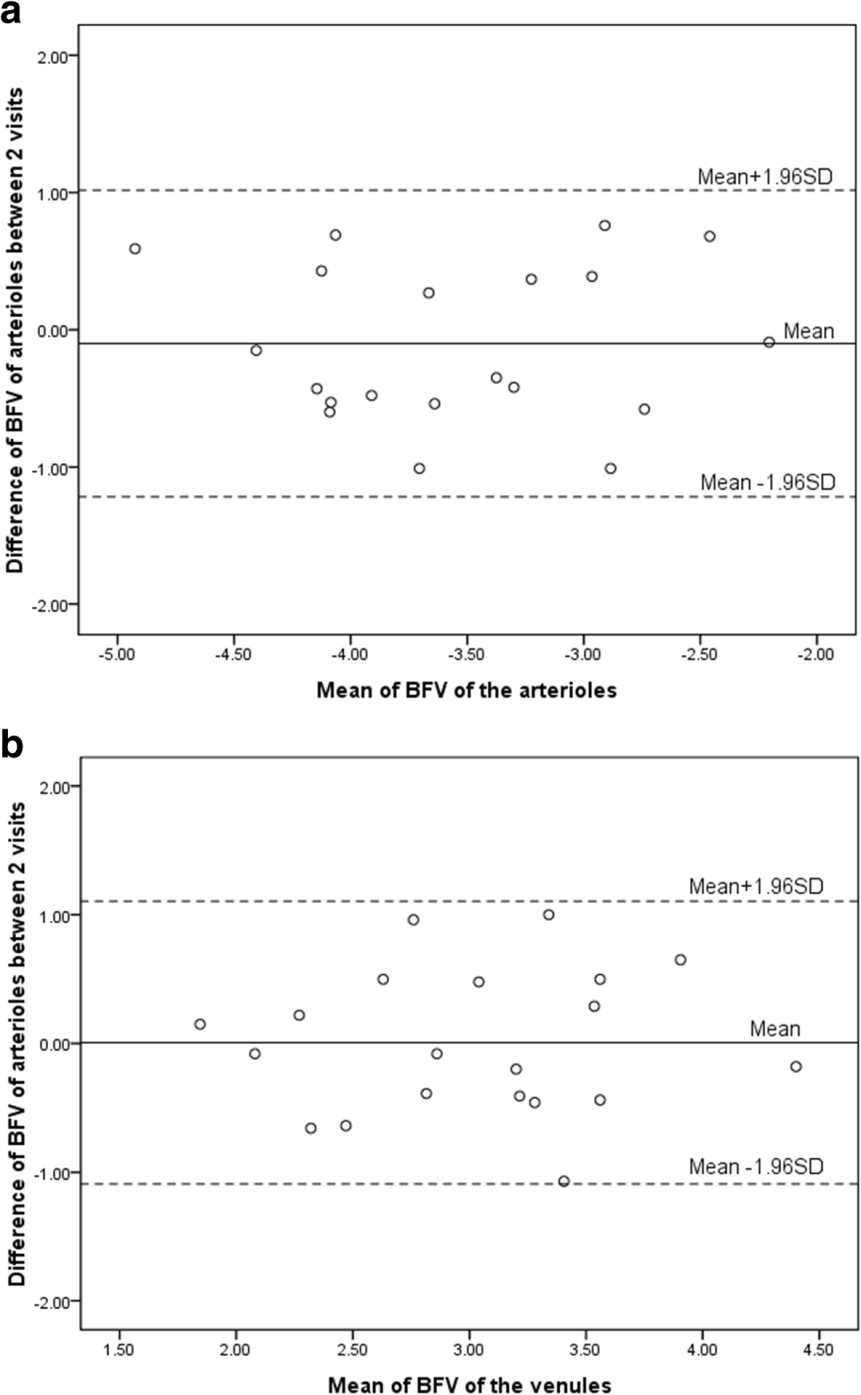
Mean value of BFV and vessel segments for two visits. Mean values of BFV in arterioles and venules showed no significant difference between visits (*P* > 0.05, **a**). Mean values of vessel segments in arterioles and venules are shown between visits (*P* > 0.05, **b**). BFV = blood flow velocity

The demographics of the study group are listed in Table 1. Twenty normal subjects (10 males and 10 females; median age = 26 years, inter-quartile range = 21 to 32 years) were imaged. A series of images of the retinal microcirculation of 20 eyes from 20 healthy subjects were successfully acquired using RFI (Fig. 1). The mean BFV of the arterioles was 3.6 ± 0.8 mm/s and 3.5 ± 0.8 mm/s in the first and second visits (Fig. 2, *P* > 0.05). The mean BFV of the venules was 3.0 ± 0.7 mm/s and 3.0 ± 0.7 mm/s in the first and second visits. There were no significant differences in vessel segments in both arterioles and venules between visits (Fig. 2, P > 0.05). The CCC of BFV was 0.72 in the arterioles and 0.67 in the venules and the CV was 10.8% in the arterioles and 11.0% in the venules. The CCC and CV of the physiological parameters are also listed in Table 2.

**Table 1:** Demographics of the study group

Characteristic	Mean	Range
Age(years)	26 ± 3	21 **—** 32
% female	50	NA
% right eye	40	NA
Refractive error (D)	- 2.5	- 6 **—** 0

**Table 2:** summary of inter-visit variability in healthy young subjects

Characteristics	1st Visit	2nd Visit	*P*	CCC	CV
	Mean ± SD	Mean ± SD			
BFV of arterioles (mm/s)	3.6 ± 0.8	3.5 ± 0.8	0.44	0.72	10.8%
BFV of venules (mm/s)	3.0 ± 0.7	3.0 ± 0.7	0.96	0.67	11.0%
No. of the segments of arterioles	30 ± 7	29 ± 7	0.43	0.35	16.9%
No. of the segments of venules	32 ± 9	32 ± 10	0.83	0.64	14.6%
Systolic blood pressure (mmHg)	109.5 ± 12.1	109.9 ± 12.6	0.81	0.82	3.7%
Diastolic blood pressure (mmHg)	65.2 ± 8.1	67.6 ± 10.0	0.56	0.69	6.2%
Heart rate (beats per min)	66.6 ± 10.3	67.8 ± 10.4	0.43	0.59	6.8%
IOP (mmHg)	13.5 ± 2.9	13.3 ± 2.8	0.59	0.79	7.0%

*BFV* = blood flow velocity; *IOP* = intraocular pressure; *CCC* = concordance correlation coefficient; *CV* = coefficient of variance

**Fig. 3:**
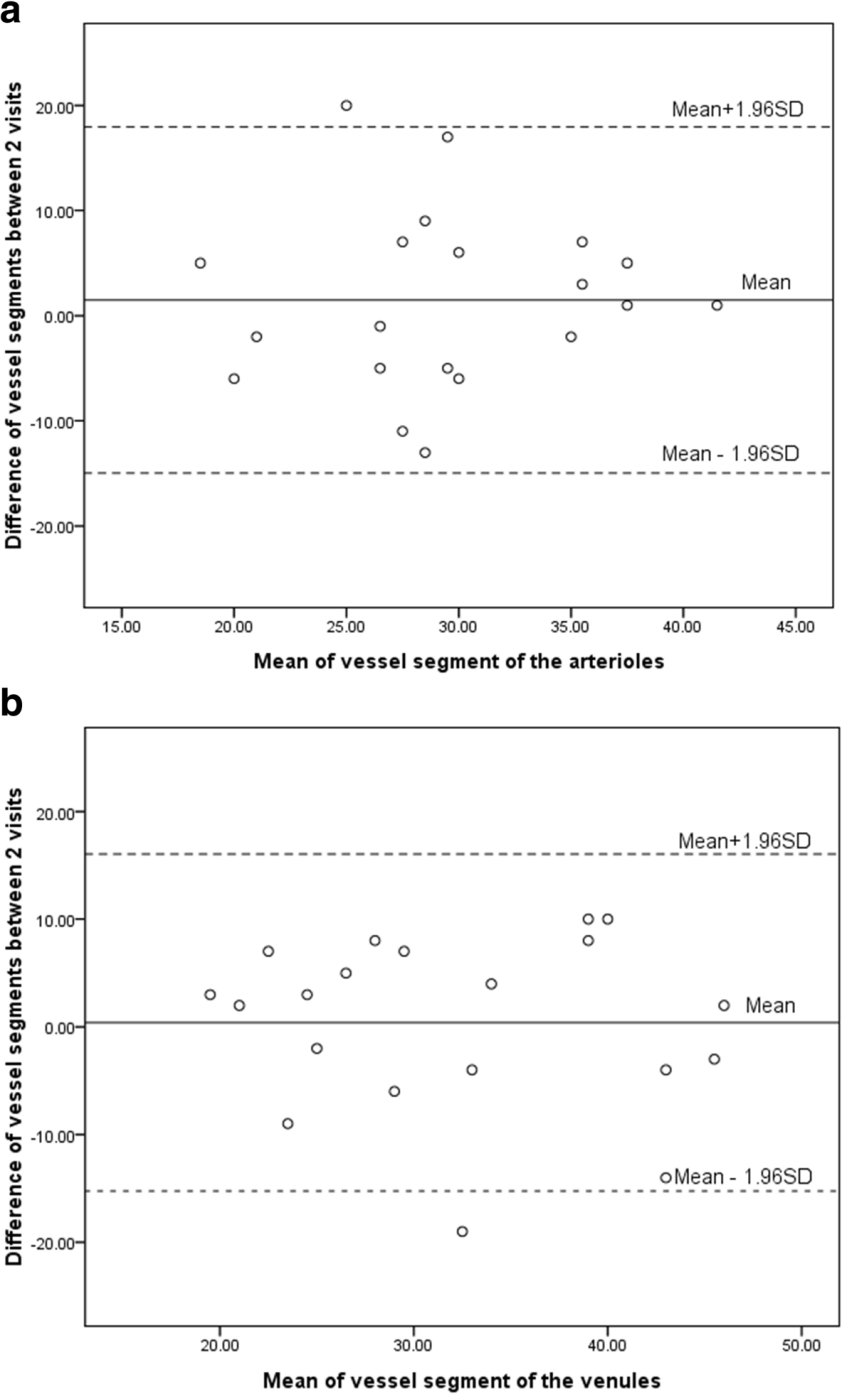
Limit-of-agreement plots of inter-visit repeatability for BFV. **a**: limit-of-agreement showing the repeatability of the BFV of arterioles. **b**: limit-of-agreement showing the repeatability of the BFV of venules. BFV = blood flow velocity

**Fig. 4:**
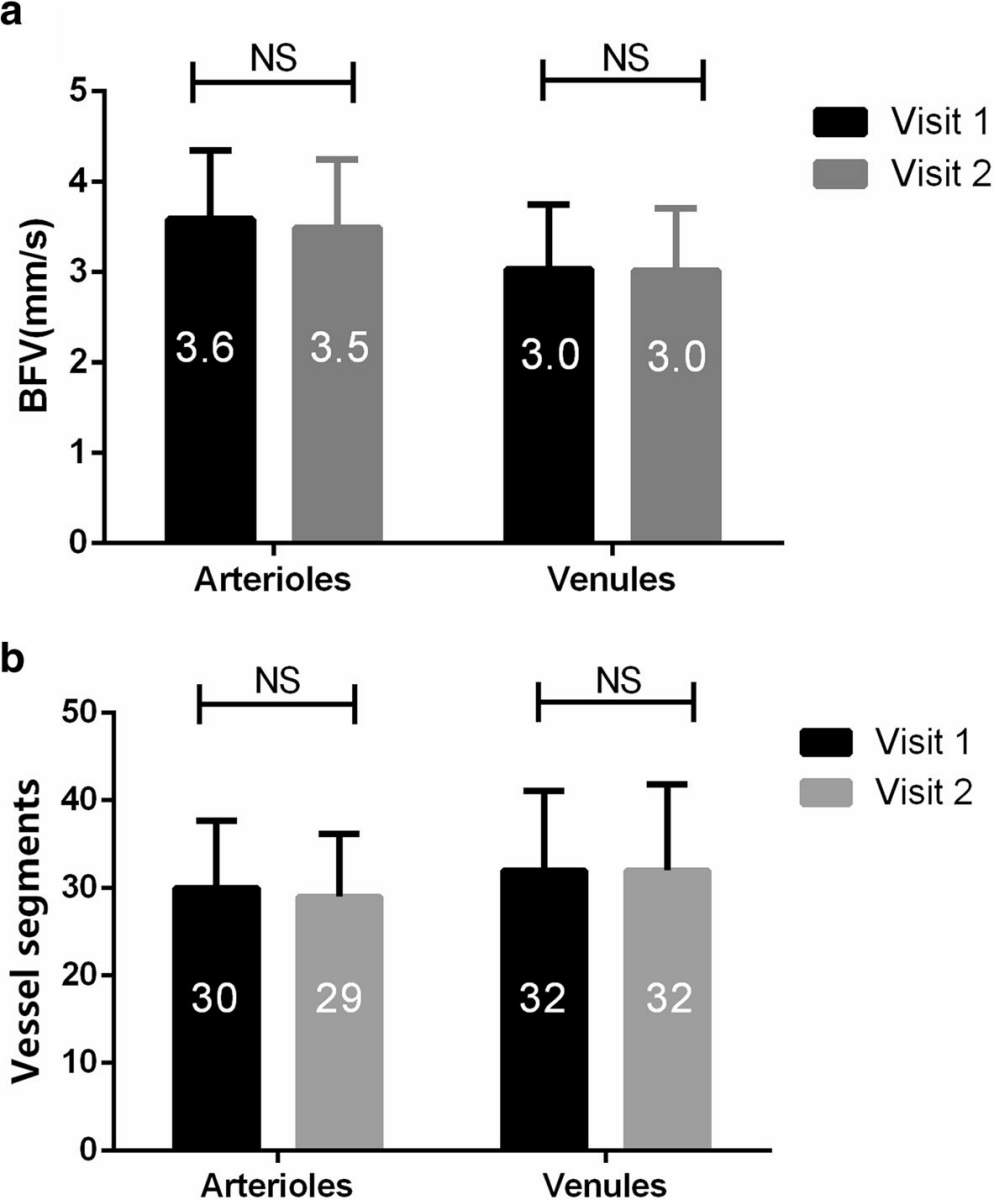
Limit-of-agreement plots of inter-visit repeatability for vessel segments. **a**: limit-of-agreement showing the repeatability in vessel segments of arterioles. **b**: limit-of-agreement showing the repeatability in vessel segments of venules. BFV = blood flow velocity

Limits of agreement plots for inter-visit differences in the mean BFV with 95% upper and lower limits of repeatability are presented in Fig. 3. The 95% limits of agreement spanned from − 1.22 to 1.02 in the mean BFV of arterioles and from − 1.10 to 1.10 in the mean BFV of venules. The limits of agreement for the mean inter-visit vessel segments were similar in arterioles (95% upper and lower limits of agreement, − 15 to 18) and venules (− 15 to 16) (Fig. 4). IQR (Inter-Quartile Range) of BFV in arterioles and venules were 0.99 and 0.92. IQR of vessel segment of arterioles and venules were 11.75 and 11.00.

A significant difference was found between males and females BFV measurements in the venules (*P* < 0.05), the CCC of delta was 0.29 and CV was 69.2%, but no significant difference in BFV measurements was found in the arterioles between males and females (*P* > 0.05),the CCC was 0.20 and CV was 62.9%. Spearman correlation analysis showed no significant association between the differences in the BFVs between visits and the differences in SBP, DBP, HR, IOP and vessel segments, as well as age, sex, and eye (P > 0.05).

## Discussion

To the best of our knowledge, this study is the first to determine the inter-visit variability of BFV with a relatively large field of view using RFI. The inter-visit variability is compatible with a previous study conducted by Burgansky-Eliash et al. who examined 5 subjects on two different days [19]. The inter-visit variability of this previous study used a small field of view (4.3 × 4.3 mm^2^ at a setting of 20 degrees), and the interclass correlation coefficient was 0.74 on average, assuming the average value was from arterioles and venules [19]. Although the correlation coefficient can be regarded as a good correlation in the measurement of the dynamic blood flow system, the information of the measurement variability will need to be considered in the study design, which requires repeated measurement of BFVs, together with the intra-visit (i.e., same visit) and inter-eye variability. Another consideration for the study design is the setting of the field of view in the RFI system. A large field of view involving a 35-degree setting in RFI has a relatively deeper focus depth, which makes it easier to focus on measuring more vessel segments. A small field of view such as that involving a 20-degree setting has a more detailed view of the smaller vessels due to the relatively higher lateral resolution. However, a small field of view requires a highly stabilized eye and sharp focus. Although both settings were used in previous studies, the ICC of the 35-degree setting appears to be comparable to the inter-visit ICC of the 20-degree setting (0.74) reported by Burgansky-Eliash et al. [19].

The inter-visit variability measured in the present study appeared to depend on the intra-visit and inter-eye variability [18, 19] (Table 3). Burgansky-Eliash et al. studied the intra-visit variability in 20 participants using the 20-degree FOV and reported a coefficient of variance (CV) of 7.5%, which is slightly better than our inter-visit measurements. The additional variability may be due to the “real” variation in blood flow velocity [19]. Chhablani et al. compared the inter-session variability of individual vessel segments in 15 normal human subjects in the same visit [18]. The inter-session concordance correlation coefficient (CCC) of BFV in the individual vessel segments was 0.97, and the CV of BFV was 10.9% [18]. In our study as well as the one by Burgansky-Eliash et al. [19], averaged velocity measurements were used to calculate the measurement variability, while Chhablani et al. only calculated the BFVs in 5–6 vessels in each eye [18]. The limited number of measurements conducted in the selected vessels appeared to result in unexpectedly low BFVs (3.16 mm/s in arterioles and 3.15 mm/s in venules), which are greatly below the normality level of previous studies [13]. As the majority of these previous studies used the average BFV [13], applying the variability of the RFI measurements while using the average results may provide more realistic information for the calculation of the sample size.

**Table 3:** Major published of repeatability evaluation of RFI in normal subject

Authors	Design	N	FOV	Characteristics	Effect	Repeatability
					Mean ± SD	
Chhablani et al. 2013 [18]	Comparing different sessions of the same eye in the same visit	15	35	BFV of arterioles(mm/s)	3.16	CCC: 0.97
				BFV of venules(mm/s)	3.15	CV: 10.9%
				No. of the segments of arterioles	5.4	
				No. of the segments of venules	6.1	
Burgansky-Eliash et al. 2012 [19]	Intra-visit variability Inter-visit variability on different days	20	20	BFV of arterioles(mm/s)	4.2 ± 0.9	Intra-visit CV: 7.5% ± 3.7% Inter-visit ICC variability: 0.74
		5		BFV of venules(mm/s)	3.3 ± 0.8	
				No. of the segments of arterioles	16 ± 6	
				No. of the segments of venules	16 ± 5	

*BFV* = blood flow velocity; *FOV* = field of view; *CCC* = concordance correlation coefficient; *CV* = coefficient of variance; *ICC* = interclass correlation coefficient

Many factors may contribute to the variability in BFV measurements, including the inherent measurement errors of the system and variability of human hemodynamics. Regarding the measurement errors of the imaging system, manual selection of all measurable second and tertiary branches of the retinal vessels and an arbitrary cut-off of 0.45 (rate of standard deviation and mean) for removing so-called unreliable measurements may mainly contribute to the variability regardless of the intra-session, inter-visit or inter-eye measurements [13, 18, 19]. Concerning the subjects, blood pressure, heart beat and intraocular pressure account for approximately 10% of the inter-visit variability, with the CCCs ranging from 0.59 to 0.82, which may influence the BFV measurements. The narrow range may be due to the auto-regulation and individual variation in blood flow and contribute to the low CCC in retinal BFV. However, no correlation was established in the present study, indicating that the impact may be multi-factorial and subject to the individual variability of the study subjects. Future studies with a large sample size may confirm this speculation. Since the variabilities of the BFVs are random, the mean BFVs may not have been impacted as shown in the present study. Previous studies have demonstrated that BFV measurements may be used in the clinic and research to study various systemic, cerebral and ocular conditions [13].

Our findings should be considered in light of several limitations. First, we did not make a comparison between both eyes on the same day. Second, measurements were not performed in healthy subjects of all ages, and no elderly subjects and patients with diseases were recruited, which limited the interpretation of the results. Third, we only measured blood pressure and heart beats during visits but not in each RFI measurement session, which may have contributed to the measurement variability. Further monitoring of the blood pressure and heart beat may provide further information.

## Conclusions

In summary, the inter-visit variability using the retinal function imager (RFI) with a large field of view appears to be good and comparable to previously reported intra-visit and inter-eye variabilities. The information on variability in the direct measurement of blood flow velocity in small retinal vessels may assist in the design of future studies requiring repeated measurements.
